# A Review of Nontraditional Biomanipulation

**DOI:** 10.1100/tsw.2008.144

**Published:** 2008-12-14

**Authors:** Xia Zhang, Ping Xie, Xiaoping Huang

**Affiliations:** ^1^ LED, South China Sea Institute of Oceanology, Chinese Academy of Sciences, Guangzhou 510301, China; ^2^ Donghu Experimental Station of Lake Ecosystems, State Key Laboratory for Freshwater Ecology and Biotechnology of China, Institute of Hydrobiology, Chinese Academy of Sciences, Wuhan 430072, China

**Keywords:** cyanobacterial blooms, filter-feeding fish, nontraditional biomanipulation, silver and bighead carp, enclosures, large-scale observation

## Abstract

The aim of this review is to identify problems, find general patterns, and extract recommendations for successful management using nontraditional biomanipulation to improve water quality. There are many obstacles that prevent traditional biomanipulation from achieving expectations: expending largely to remove planktivorous fish, reduction of external and internal phosphorus, and macrophyte re-establishment. Grazing pressure from large zooplankton is decoupled in hypereutrophic waters where cyanobacterial blooms flourish. The original idea of biomanipulation (increased zooplankton grazing rate as a tool for controlling nuisance algae) is not the only means of controlling nuisance algae via biotic manipulations. Stocking phytoplanktivorous fish may be considered to be a nontraditional method; however, it can be an effective management tool to control nuisance algal blooms in tropical lakes that are highly productive and unmanageable to reduce nutrient concentrations to low levels.

Although small enclosures increase spatial overlap between predators and prey, leading to overestimates of the impact of predation, microcosm and whole-lake experiments have revealed similar community responses to major factors that regulate lake communities, such as nutrients and planktivorous fish. Both enclosure experiments and large-scale observations revealed that the initial phytoplankton community composition greatly impacted the success of biomanipulation. Long-term observations in Lake Donghu and Lake Qiandaohu have documented that silver carp (*Hypophthalmichthys molitrix*) and bighead carp (*H. nobilis*) (two filter-feeding planktivorous species commonly used in management) can suppress Microcystis blooms efficiently. The introduction of silver and bighead carp could be an effective management technique in eutrophic systems that lack macrozooplankton. We confirmed that nontraditional biomanipulation is only appropriate if the primary aim is to reduce nuisance blooms of large algal species, which cannot be controlled effectively by large herbivorous zooplankton. Alternatively, this type of biomanipulation did not work efficiently in less eutrophic systems where nanophytoplankton dominated.

## POTENTIAL FOR NONTRADITIONAL BIOMANIPULATION TO CONTROL BLUE-GREEN BLOOMS

### Limitations of Traditional Food Web Biomanipulation

Trophic cascades have been described as indirect top-down effects on two or more trophic levels[1,2] that have the potential to drive regime shifts in aquatic systems[3,4]. Concepts of trophic cascades[2] and biomanipulation[5] have linked community structure to biogeochemical processes in pelagic ecosystems, and have prompted a diversity of comparative and experimental studies. The point of classical biomanipulation is an increase in the abundance and size of zooplankton (mainly large *Daphnia* species), which results in enhanced grazing pressure on phytoplankton and, ultimately, clear water of lakes[6]. Biomanipulation involves eliminating certain fish species or restructuring the fish community to favor the dominance of piscivorous fish instead of planktivorous fish. There are at least five methods used singly or in combination, and those are stocking of piscivores, fish winter kill, rough fish removal, use of fish poisons, and water level drawdown[7]. Although the potential of planktivores to reduce zooplankton biomass is evident[8,9,10], the ability of zooplankton to control phytoplankton biomass in lakes has been found to be more variable[8,11,12,13,14,15]. Moreover, as the peculiar structures and processes involved are highly variable, it is often difficult to achieve the predicted outcome of a biomanipulation experiment quantitatively. There are many conditions that can impact the success of biomanipulation: (a) planktivorous fish must be removed repeatedly or else they can become re-established; (b) in terms of sufficiently low “thresholds”[16] of phosphorus loading, grazers can reduce chlorophyll concentrations; (c) even when the external phosphorus has been reduced considerably, and resistance to improvement is often observed, phosphorus can remain high because of phosphorus release from the sediment pool[17]; (d) macrophytes are essential for stablizing clear water states[18,19,20]. Benndorf et al.[21] argue that biomanipulation should be restricted to certain types of lakes that meet a set of conditions in order to be successful for a long time period.

## BIOMANIPULATION WITH FILTER-FEEDING PLANKTIVOROUS FISH: NONTRADITIONAL BIOMANIPULATION

Parallel to traditional biomanipulation, some ecologists have attempted to explore another methodology applicable for hypereutrophic lakes with bloom-forming cyanobacteria and absent grazing pressure from zooplankton. Several previous studies have shown that large-bodied zooplankton generally have higher mass-specific grazing rates on algae than small ones[22]. However, many studies have found large declines of zooplankton biomass in summer as a consequence of a decline in large *Daphnia* concomitant with the occurrence of cyanobacterial blooms[23,24,25]. Cyanobacteria can exert a negative impact on zooplankton growth and composition by interference or poor food quality and can also be toxic[26]. On the other hand, large-bodied zooplankton are absent in numerous eutrophic lakes where zooplankton-phytoplankton interaction is decoupled and nuisance algal blooms often occur in summer. In the above conditions where nutrients cannot be reduced to a certain low level, an alternative biomanipulation approach, based on the direct control of undesirable bloom-forming blue-greens by filter-feeding planktivorous fish, appears to be more effective[27,28,29,30,31,32,33,34,35]. Filter-feeding fish feed on phytoplankton by engulfing or sucking a volume of water containing prey items into the buccal cavity. This feeding behavior is not visually directed at individual prey and, therefore, filter-feeding fish may capture more than one prey at a time[31]. Silver carp is an obligating filter-feeding planktivore, consuming mainly phytoplankton, zooplankton, and/or suspended small particles[31,36]. Most literature cites the bighead as being predominantly zooplanktivorous[37,38,39]. However, this species is opportunistic and when concentrations of zooplankton are low, it may feed on phytoplankton and detritus[40].

In contrast to the introduction of piscivores, stocking planktivorous fish to eliminate blue-green algae has made progress in various enclosure and whole-lake experiments. While Starling et al.[41] listed 14 successful studies of one planktivorous species (silver carp), others found no evident or even reversed effects[32,42,43,44]. However, the controversial nature of the use of planktivorous fish in water quality management undoubtedly stems from observations that stocking silver carp, for example, usually increases rather than decreases phytoplankton biomass[29,38,45]. Numerous studies have explored two key factors that contribute to this phenomenon. First, gut content analysis and laboratory feeding experiments demonstrated that silver carp efficiently consume net phytoplankton and large zooplankton, but not algae <10 µm in largest dimension[46]. Second, silver carp are omnivorous rather than phytoplanktivorous. When plankton concentrations are sufficiently high, silver carp probably behave as pelagic omnivores, but when plankton concentrations are low, silver carp become epibenthic browsers[47].

It is well known that planktivorous fish can affect the structure and dynamics of phytoplankton communities through direct predation, zooplankton grazing, and nutrient recycling mechanisms. Evidence for the role of nutrient recycling by fish derives from two types of studies[48]. The first calculates the total nutrient uptake rate by phytoplankton. The conclusions of such studies are mixed with respect to whether recycling by planktivorous fish is a major nutrient source for the phytoplankton[49,50,51]. At best, these studies provide only indirect evidence for the role of nutrient recycling by fish in the trophic cascade response[52]. Alternatively, a few studies isolated the influence of nutrient recycling by fish from that of zooplankton grazing[53,54,55]. These studies tend to support the view that nutrient recycling by fish is an important mechanism driving the response of phytoplankton biomass to fish manipulation. Nevertheless, the confounded mechanisms of recycling and grazing have not been separated at the whole-lake scale[56].

## Different Nutrient Fluxes Involved in Phytoplankton Assemblage Dynamics

The enclosure experiments in Lake St. George were a good example of the enhancement of algal populations by nutrients recycled from fish[57]. In this study, planktivorous fish did not affect the biomass or body size of zooplankton, but increased nutrient availability through recycling by fish. This observation supported the findings of Vanni and Findlay[58], Drenner et al.[59], Threlkeld[60], Schindler[53], and others in highlighting the importance of fish as sources of nutrient regeneration. It was well understood that small-celled algae benefit from both reduced grazing and nutrient supply in the presence of fish compared with the control treatment. Small algal cells provide more potential competitors for nutrients than large cells[61]. Consequently, these small-sized species should be able to increase greatly and the system would shift towards an algal assemblage dominated by small ones[43].

However, those with the opposite view demurred that the contribution of phosphorus from fish excretion is insignificant in natural ecosystems[49]. On the other hand, the importance of zooplankton excretion to phytoplankton communities is a subject of debate. The extent of algae response to fish-mediated nutrient recycling effects and whether these effects are more important than those through zooplankton herbivory is uncertain. Contrary to results of an experiment conducted by McQueen et al.[57], planktivorous fish reduced zooplankton biomass and body size in another enclosure experiment[62] where it was determined that phosphorus was recycled by both zooplankton and fish, but the primary nutrient source was zooplankton. It has been found that smaller zooplankters have higher mass-specific excretion rates per unit of biomass than fish, but this was not enough to offset the reduction in total zooplankton biomass caused by fish predation[62]. It was supposed that planktivorous fish may indirectly increase nutrient recycling rates by zooplankton, if total zooplankton biomass is not reduced by fish. Persson[63] observed that phytoplankton increased only when exposed both to nutrients released by fish and to reductions in zooplankton biomass, but not when exposed only to nutrients or only to zooplankton reduction. Therefore, how phytoplankton will respond to fish should depend on the relative strength of the different mechanisms by which fish can affect phytoplankton and on how total zooplankton biomass responds to fish[55].

On the other hand, some algal species, especially related to bloom-forming species (e.g., *Microcystis* spp. or *Aphanizomenon* spp.) can uptake phosphorus during passage through fish guts[64,65]. Lewin et al.[66] detected that *Microcystis* with mucilage was almost undamaged and showed high viability after passage through roach guts, and live colonies had a significant higher radioactivity than dead ones. They suggested that *Microcystis* with mucosal covering was protected against digestion and can directly use the phosphorus supplied in the fish guts during passage. Thus, Kolmakov and Gladyshev[67] considered planktivorous fish as “living cultivators” for some bloom-forming colonial and filamentous cyanobacteria, enhancing their growth and photosynthetic activity. Results of viability of *Microcystis* ejected in the fecal matter of tilapia, silver and bighead carp using filtered lake water as medium during 4-days incubation revealed about a seven- to eightfold increase of cyanobacterial biomass[68]. However, all of the experiments above were conducted *in vitro*, which differed greatly from natural conditions. Colonies of *Microcystis* were broken down to single cells during passage through fish intestines[69]. Feces contained small colony or single cells of cyanobacteria settled down to the bottom and fecal nutrients are usually not immediately available to phytoplankton. The process of recruitment of *Microcystis* from sediment is regulated by several interactions involved with various factors, not only by capturing “additional” nutrients. Perhaps in natural environmental conditions, this process is exaggerated, as algal biomass is a balance of beneficial nutrient recycling affected by fish and negative effects of herbivorous fish on algae.

## Predation Suppression from Upper Trophic Levels

Filter-feeding fish, such as silver carp, can suppress zooplankton through direct grazing or decreasing the algal biomass of available forms to zooplankton[38]. Predation pressure by filter-feeding fish mostly shifts the zooplankton community structure toward increasing densities of small-sized or evasive calanoid copepods[30,31]. In conditions where macrozooplankton was abundant, suppression by fish enhanced the competitive advantages of fast growing nano- and picophytoplankton, released from zooplankton grazing pressure. The magnitude of this indirect enhancement of small algae highly depends on the trade-off between fish-mediated suppression of its consumers (zooplankton) and its competitors (net phytoplankton)[70]. Whereas in eutrophic subtropical lakes, characterized by a weak relationship between net phytoplankton and microzooplankton, silver carp are able to control net phytoplankton and increases of nanoplankton abundance do not occur[43]. Lazzaro[71] demonstrated that zooplankton suppression was a function of filter-feeding fish biomass, which was consistent with numerous observations[32,38,47,72]. Domaizon and Dévaux[43] proposed a threshold density of silver carp of 8 g $m^{-3}$, below which negative effects on herbivorous zooplankton should be minimized and beneficial effects on phytoplankton could be efficient. Fukushima et al.[44] demonstrated that, in the presence of both silver carp and crustaceans, the top-down forces of the latter could overwhelm the filter-feeding efficiency of silver carp, at least when water temperatures are too low to induce cyanobacterial blooms. However, in the case when colonial species were dominant, total phytoplankton was reduced significantly by silver carp, as silver carp is a more efficient grazer on net phytoplankton than crustaceans due to size-selective grazing[73]. Consequently, Radke and Kahl[27] concluded that use of silver carp in biomanipulation is only appropriate if the primary aim is to reduce nuisance blooms of large algal species that cannot be effectively controlled by large herbivorous zooplankton. Zhu and Deng[74], using isotope techniques, found the assimilation rate of silver carp fed with *M. aeruginosa* to be 35–48%. Although ingestion and growth was supposed when silver carp were exposed to toxic strains of *Microcystis* in the laboratory[75], Chen et al.[76] discovered that silver carp could effectively ingest toxic *Microcystis* cells up to 84.4% of total phytoplankton, but showed fast growth in Lake Taihu (from 141 to 1759 g in 1 year in mean weight).

To understand how planktivorous fish, especially common managed silver and bighead carp, interact with other components in food webs and thus affect plankton dynamics and water quality, we present a short historical background behind the development of nontraditional biomanipulation. Derived from various enclosures and large-scale experiments, we aim to examine whether it can be a successful alternative to accelerate lake recovery. Because theory predicts variable results, multiple field trials are needed in lakes that differ in catchment geochemistry, littoral zone development, trophic state, and food web composition[77,78,79]. Interpretational problems arise because field experimentation often requires a reduction in spatial and temporal scale below that of the system of interest[80,81,82]. Although manipulations at the scale of entire communities can obviate some of these problems, such experiments will always be rare in general, and logistically impossible in many systems. Drenner[83] suggested that microcosm and whole-lake experiments had revealed similar community responses to major factors regulating lake communities, such as nutrients and planktivorous fish.

## SMALL-SCALE EXPERIMENTS: A ROUGH DRAWING OF THE THEORY

Summed from 30 field enclosure (microcosm or mesocosm) experiments managed on silver or bighead carp conducted in various water systems since 1975, the average success rate of nontraditional biomanipulation is about 63% (19 of 30 case studies[29,33,38,42,44,68,70,100,103,104,105,106,107,108, 109,112]) and nine experiments indicate fish did not significantly suppress phytoplankton[27,38,43,47, 101,108,110,113], and planktivorous fish have no effects in other studies[44,89] (Table 1). Here we consider management to be successful if total algal biomass is suppressed after fish introduction. Numerous studies explored four crucial factors that probably explain the inconsistent results: (a) the stocking level of fish[43], (b) the initial zooplankton species pool[41,44], (c) the initial phytoplankton species pool[68], and (d) temperature conditions[44]. Are these conflicting results owing to different levels of stocking fish? We listed stocking levels ranging from 0.74 to 480 g $m^{-3}$ in all of 30 enclosure or pond experiments (Table 1). We observed that there was no increasing relationship between stocking density and algal reduction. However, Tang et al.[73] suggested that neither fish biomass nor nutrients were important in determining whether the effect on phytoplankton biomass was positive or negative, because fish biomass and nutrients largely overlapped within these two effects. They deduced that the initial phytoplankton composition and the response of the plankton community to silver carp were mostly related to successful management. We found that all of the 19 experiments biomanipulated successfully have in common the fact that they were performed under eutrophic or hypereutrophic conditions where algal blooms ought to be suppressed, and large or colonial algae (mainly cyanobacteria) were the predominant phytoplankton forms prior to fish stocking. Opuszynski[45] investigated the food of the silver carp in relation to environmental conditions, found distinct food selectivity, and came to the conclusion that the activities of this fish could not be compared to a plankton net mechanically straining out particles above a given size. Food consumed by silver carp is strongly dependent on food availability in the environment[36,44,84]. If the original phytoplankton population is dominated by net phytoplankton, introduction of silver carp typically reduced the total chlorophyll *a*. However, if the phytoplankton is dominated by nanoplankton, introduction of silver carp could very easily cause an increase in chlorophyll *a*.

There is widespread consensus that biomanipulation probably has a much higher success rate in shallow than in stratified deep lakes[85,86]; whether this feature is applicable to nontraditional biomanipulation is unclear. Shallow enclosures may increase spatial overlap between competitors and between predators and prey, leading to overestimates of the impact of both predation and interference competition[87]. We find only one enclosure experiment conducted in a deep reservoir[27]. Planktivorous fish increased total chlorophyll *a*, but decreased zooplankton biomass and body size. The small size of algae in the fraction <10 μm, which comprised 99% of the total cell number, may account for the failure of biomanipulation[27]. Therefore, either in shallow or deep waters, the original phytoplankton populations may be the key as to whether or not the management will be successful. Therefore, prior to implementing this biomanipulation technique on a large scale, it is critical to assess the threshold of stocking density available to suppress blooms in an experimentally partitioned area. Lakes under conditions of high temperature, absent of large

**TABLE 1**
**Experiments and Field Studies Examining the Effects of Two Filter-Feeding Fish Species on Phytoplankton Biomass, Zooplankton Abundance, and Total Phosphorus (TP) Content***

| Species | Ref. | System | Efficient Stocking Biomass (g m$^{-3}$) | Carp Effects** | | |
|---|---|---|---|---|---|---|
| | | | | Phytoplankton Biomass*** | Zooplankton Abundance | TP |
| Silver carp | [29] | Tanks with refuge | ---------- | Decreased | No effect | ---------- |
| | [33] | Enclosures (1990) (S+B) | 12–84.5 | Decreased | ---------- | ---------- |
| | | Enclosures (1992) | 21–33 | Decreased | ---------- | ---------- |
| | [42] | Enclosures | <27.2 | Decreased | Increased | No effect |
| | [44] | Enclosures (summer) | 10 | Decreased | Decreased | Decreased |
| | [68] | Enclosures | 480 | Decreased | ---------- | No effect |
| | [70] | Fiberglass tanks | 41 | Decreased | No effect | No effect |
| | [100] | Enclosures | 30–90 | Decreased | Decreased | ---------- |
| | [103] | Enclosures | 5.58–9.08 | Decreased | Decreased | Increased |
| | [105] | Ponds | 1.56 | Decreased | ---------- | ---------- |
| | [106] | Enclosures (1999) | 46.4–126.4 | Decreased | Decreased | No effect |
| | [107] | Enclosures | 9.1–78.8 | Decreased | ---------- | ---------- |
| | [108] | Aquaria | 30 | Decreased | Decreased | Decreased |
| | [109] | Enclosures with *Potamogeton crispus* | 75 | Decreased | Increased | Decreased |
| | [112] | Enclosures | 55 | Decreased | Decreased | No effect |
| | [27] | Enclosures | 10 | Increased | Decreased | Increased |
| | [43] | Mesocosms | 8 | Increased | Decreased | ---------- |
| | [47] | Aquaculture ponds | 0.74 | Increased | Decreased | ---------- |
| | [101] | Ponds with common carp | 16.2–29.4 | Increased | Decreased | ---------- |
| | [110] | Enclosures | >76 | Increased | ---------- | ---------- |
| | [113] | Ponds with common carp and small indigenous fish species (SIS) | 4.6 | Increased | ---------- | ---------- |
| | [44] | Enclosures (autumn) | 3.1–15 | No effect | No effect | Increased |
| | [89] | Ponds with catfish | 0.65–2.16 | No effect | ---------- | ---------- |
| Bighead carp | [33] | Enclosures (1989) | 365 | Decreased | Decreased | ---------- |
| | | Enclosures (1992) | 69–75 | Decreased | Decreased | ---------- |
| | [68] | Enclosures | 432 | Decreased | ---------- | No effect |
| | [104] | Ponds | 60–97 | Decreased | Decreased | ---------- |
| | [38] | Ponds with catfish and grass carp | 2.5 | Increased | Decreased | ---------- |
| | [108] | Aquaria | 30 | Increased | Decreased | Decreased |
| | [33] | Lake Donghu (S+B) | 46–50 | Decreased | Decreased | ---------- |
| | [34] | Lake Qiandaohu, China | 1.5–7.4 | Decreased | ---------- | Decreased |
| | [35] | Pens in Lake Taihu (S+B) | 40 | Decreased | Decreased | No effect |
| | [102] | Netofa Reservoir, Israel (S+B) | 0.05–0.64 | Decreased | Decreased | ---------- |
| | [111] | Lake Yuehu, China (S+B) | <9.8 | Decreased | ---------- | Increased |

*Table 1 continues*

**TABLE 1 continued**

| | | | | | |
|---|---|---|---|---|---|
| [36] | Lake Kinneret, Israel | $11 \times 10^6$ fingerlings per lake (1969–1981) | Increased | Decreased | ---------- |
| [93] | Saidenbach Reservoir, Germany | 300 kg $ha^{-1}$ | Increased | Decreased | ---------- |

* Shaded rows indicate large-scale or whole-lake studies of silver and (or) bighead carp. No effect = no significant effect of the presence of fish; ---------- = Not evaluated.

** Effects reported are the conclusions of the authors or our interpretation of trends apparent in the data.

*** Biomass was measured as chlorophyll, fluorescence, or calculated based on counts and phytoplankton size.

cladocerans, which favored blue-greens, could rely on nontraditional biomanipulation for long-term maintenance. Enclosure experiments can be a valuable tool when properly scaled for the study of particular mechanism, interactions, or flux rates, but may be misleading when results are extrapolated to entire systems[88].

## LONG-TERM WHOLE-LAKE OR LARGE-SCALE EXPERIMENTS ARE REQUISITE TO EXAMINE EFFICIENCY OF MANAGEMENT

Although silver and bighead carp may successfully control cyanobacterial blooms, their other ecological influences on accommodated ecosystems are unclear. Silver and bighead carp are native to eastern Asia. Since the 1960s, silver and bighead carp have been introduced worldwide and have been cultured for centuries in mixed cultures with other fish species, increasing total yield due to utilization of primary production[33]. Using these two species as a means of controlling blooms of algae, aquaculture ponds and sewage lagoons for improving water quality have developed since the 1970s. Because silver carp is a lean and extremely bony fish, its market value in some parts of the world is rather low. In the U.S., many fishermen do not primarily target bighead and silver carp because the price received is low ($0.10–$0.15/lb). Instead, they fish for bighead and silver carp when other species or opportunities are unavailable. On the other hand, Asian carp is not native in many countries and is considered a nuisance species[89]. It appears that species-poor fish communities favor the establishment of non-native populations, and are more vulnerable to invaders because alien species are less liable to be out-competed or predated upon by indigenous species[90]. Impacts of exotic fish on new environments can range from mild to catastrophic and include habitat alteration, trophic structure alteration, and hybridization[91]. Silver and bighead carp have been added to the list of injurious wildlife since 2007 in order to prevent their further introduction and invasion into natural waters of the U.S., and to protect native fishes, the survival and welfare of wildlife and wildlife resources, and the health and welfare of humans (Fish and Wildlife Service of the United States, 2007). An "invasive species" is defined by *Executive Order* 13112[92] of 1999 as a species that is (1) non-native (or alien) to the ecosystem under consideration and (2) whose introduction causes or is likely to cause economic or environmental harm or harm to human health. Invasive species typically have high reproductive rates, disperse easily, and can tolerate a wide range of environmental conditions. Because silver and bighead carp have survived and have become established in river systems, there is serious concern that these species will further expand their ranges, and it is highly likely that they can establish reproducing populations beyond riverine environments and into lakes. Consequently, fish introduction and stocking should be done in a cautious and responsible manner. Fortunately, it is relatively easy to regulate the population size of the carp since these species have been able to establish in few systems outside their native range because of the absence of suitable conditions. Silver and bighead carp require bodies of water with some current for eggs to float and develop properly, and they carry out migrations to communal spawning grounds during spring flooding. Thus, if the stocking is stopped, the fish should decline gradually. Hence, there probably is a potential for using silver and bighead carp to inhibit cyanobacterial blooms in eutrophic lakes.

Within seven observations processed in whole-lake or reservoir environments for a long term, two water systems were biomanipulated inefficiently[36,93] and five practices were considered to be successful[33,34,35,102,111] (Table 1). In the Saidenbach Reservoir, Germany, elevated densities of native planktivorous fish (roach and perch) and non-native silver carp resulted in increased nanoplankton and decreased *Daphnia* abundance and body length[93]. Helminen and Sarvala[94] detected that zooplankton biomass explained a significant portion of phytoplankton abundance during poor vendace year-class in Lake Pyhäjärvi. In these two cases, top-down forces from zooplankton on algae were even stronger than the effect of planktivorous fish on zooplankton. After adding planktivorous fish, zooplankton was depressed, then grazing on phytoplankton was reduced. Released from predation by zooplankton, small-sized phytoplankton can flourish, leading to a decrease in water transparency. This has been well documented in Lake Kinneret, Israel, following the introduction of $11 \times 10^6$ fingerlings of silver carp between 1969 and 1981[36]. In this system, zooplankton was consumed greatly, whereas nanophytoplankton was induced to proliferate[36].

However, in the five other systems, the strength of a trophic cascade was weaker and the system was dominated by large algal species. Lake Donghu, Lake Qiandaohu, Lake Taihu, and three eutrophic lakes in China all suffered from blue-green blooms, and the Netofa Reservoir, Israel, was bothered by a heavy bloom of *Peridinium*. Lake Donghu had been manipulated with silver and bighead carp since 1986. Cyanobacterial blooms were eliminated efficiently for 21 years. Compared to the plankton community patterns of 1985 and 1987, Miura[32] explored the essential factor triggering diminished *Microcystis* blooms. Planktivorous fish density was increased from 0.09 fish $m^{-2}$ (1985) to 0.11 fish $m^{-2}$ (1987), which probably reached the threshold of controlling nuisance algae[32]. Furthermore, Chen et al.[84] demonstrated that nutrient release by silver and bighead carp in Lake Donghu was lower than optimal requirements for algal production. It is suggested that in the eutrophic environment, nutrient excretion by fish is not critical for algal growth. Vanni et al.[95] discovered that if most food obtained is pelagic zooplankton, fish might be a net sink for phosphorus, and thus decrease the overall amount of phosphorus available to phytoplankton. Alternatively, if fish obtain a significant amount of their food from the littoral zone, they may be a net source of phosphorus to phytoplankton. The quantitative amount of nutrients recycled by fish can be calculated only in enclosure or laboratory experiments and is difficult to measure under natural conditions, as nutrients dynamic processes interact between each component of the food web.

As well as in Lake Qiandaohu, Liu et al.[34] considered that the most likely explanation for the fact that no bloom occurred for 3 years was the increased grazing due to the re-establishment of the two filter-feeding carp populations (1.5–7.4 g $m^{-3}$). They inferred that drastic declines of silver and bighead carp yields were mainly responsible for the cyanobacterial blooms in 1998 and 1999. In the past decades, silver and bighead carp have been the main species stocked in many Chinese lakes. Introduction of these two species to Meiliang Bay pens, part of Lake Taihu, testifies to the success of nontraditional biomanipulation during 2004 to 2005[35]. *Microcystis* biomass and microcystin concentrations in experimental pens were reduced to almost half the amount of the outer surrounding lake during summer. It was predicted that a higher stocking density of fishes (>40 g $m^{-3}$) could have an even greater effect. Li et al.[96] reported the distribution patterns and dynamics of microcystins in the liver and kidney of silver carp cultured in the same fish pens in Lake Taihu; silver carp displayed only slight pathological changes in spite of a higher accumulation of microcystins in their livers during *Microcystis* blooms. Recovery in the ultrastructures of the liver and kidney after *Microcystis* blooms was accompanied with a significant decrease or even disappearance of microcystins. It is confirmed that the planktivorous silver carp has strong resistance to toxic cyanobacterial blooms[76,97]. It has been documented that silver carp is most suitable for clearing *Microcystis* in the lake, as it is an efficient grazer of *Microcystis* and clearance rates remained fairly high, ranging from 74 to 93%[68].

Ontogenetic niche shifts and size-structured interactions of fish populations add to the complexity of lake food webs and make scientifically sound predictions of manipulation success more difficult than was previous envisaged[98]. Further research on plankton-associated food webs is required to build up a good theory applicable to local conditions. As stated by Lehman[99], “each natural or man-caused perturbation to a lake is to some degree a novel experiment because no two lakes are identical nor are weather and climate invariant.” Whole-lake experiments have also found inconsistent effects of planktivorous fish on phytoplankton and water transparency, even when the manipulations are designed to “pound the systems” to achieve lake responses. In order to check the effects of management, enclosure experiments have contributed substantially to progress in understanding complex lake food webs, which should in turn promote a higher success rate of future whole-lake experiments, as well as set directions for the development of nontraditional biomanipulation as a management tool to improve water quality. We conclude that this type of biocontrol is more likely to be successful in eutrophic systems that are dominated by nuisance blue-greens, and may be ineffective in water bodies where nanophytoplankton predominates.

## ACKNOWLEDGMENTS

We wish to acknowledge the editor and two anonymous reviewers who provided their insightful and constructive comments that improved this manuscript greatly.
